# The Dental Clinic Abroad and at Home

**Published:** 1912-12-14

**Authors:** 


### THE IDEAL SCHOOL CLINIC.

### The Dental Clinic Abroad and at Home.

The importance of paying some attention, systematically and methodically, to the teeth of school children has often been insisted upon by dentists as well as by medical men and teachers. The last have unfortunately paid less attention to this important branch of school hygiene than they might have done, but school teachers, as a class, are now doing their best by precept and example to make up for former errors of omission. In some parts of the country the question of the condition of the children's teeth has been carefully investigated by the school doctors, with the co-operation of the teachers, and some very interesting information has been obtained by those investigations. Work of this character has been much more frequently—and be it added, more systematically and scientifically—done abroad, especially in America and in Denmark, and from the facts obtained one or two conclusions may legitimately be drawn.

The first of these is that it is possible, by care and attention paid to the teeth during the school-going age of the child, to preserve a number of the teeth which would otherwise inevitably be destroyed by caries. The cause of dental caries, it is unnecessary to state, is not yet known, although it is probable that errors in diet are chiefly responsible for the onset of the disease in the vast majority of cases, while in others there is reason to believe that a hereditary predisposition underlies the condition. Be this as it may, the fact has been recognised that a great deal may be done by prophylactic measures and suitable instruction to the children in the elements of oral hygiene, to stop the spread of the disease in cases where it has not advanced very far. Extraction and conservative treatment by stopping, scaling, and fixing are necessary in most cases, and in all in which there is any evidence of dental caries the intervention, if only for systematic examination, of a qualified dentist becomes indispensable.

This has been recognised, it is satisfactory to say, in several centres which have decided to establish dental clinics in connection with already existing school clinics, or in cases where no such school treatment centres existed, as independent centres of dental treatment. In Essen a new dental clinic is to be started; at Rostock a school clinic is doing excellent work; at Goeppingen, Kirchberg, Dortmund, Hamburg, Cassel, Leipzig, Gottingen, Kiel, Charlottenburg, and other centres such dental clinics are already in existence. They treat children who are recommended for dental treatment by the school doctors. A qualified dentist, who is ranked as a head-master—that is to say, he receives the same scale of pay and is entitled to a State pension calculated on that scale—gives his whole time to such a clinic in the majority of cases. In a few half-time or part-time dentists are employed, but this system has not been found to work well, and the tendency in Germany is to eliminate the part-timer and to employ whole-timers; in this way the local authority certainly gets better work, and is in a far better position to control that work as a whole. Payment for work done on behalf of the children is exacted from the parents, who may either commute their liabilities by an annual subscription, which entitles them to send one child to the clinic at any time during the year, or pay according to the work done. The average yearly subscription per child is one mark; the average rate per child on the second basis of payment works out at about one shilling and threepence, though in some places the charges are much higher than in others. For instance, at Hamburg the charge made at the admirable school clinics for dental treatment averages a mark and a half, a third of which is paid by the parents while the remainder is debited to the local authority. The clinic itself is supported by the rates.

In the German dental clinics, which may be regarded as models, conservative treatment is the rule, extraction is discouraged, and fillings, notwithstanding the fact that they cost money, are provided wherever they are necessary. The annual cost of the clinics is therefore relatively high, and in order to make the charge per patient treated a small one a large number of children have to be treated daily. In Denmark and in England the number of fillings is comparatively small; with us the school dentist contents himself in the majority of cases with extracting the worst teeth. As a matter of fact, in the few dental school clinics that we possess the work done is by no means so good as it might be. Very often a school doctor who has had occasion to recommend a child for dental treatment on account of carious teeth finds on examination at a later period that the dentist has been content with the extraction of one of the most carious of the teeth, and that the child has not even been prescribed a mouth-wash. In some schools the laudatory custom of having "tooth-brush drills" has been initiated. In America the school nurse is supposed to organise such drills and to see that the children fully understand and appreciate the importance of daily cleaning the teeth. With us, it is to be feared, such precepts are left to be instilled by the teachers, for we have not yet progressed to the stage where the school nurse is encouraged to lecture to the children on elementary hygiene.

It is only by the systematic examination of school children that the problem of dental caries can be attacked with any hope of success so far as the school-going population is concerned. For that reason the establishment of dental school clinics is imperatively necessary. The arguments in favour of hospital treatment for cases of disease of the nose and throat, and therefore the arguments against the establishment of special school clinics to deal with these conditions, are far more potent than those which can be advanced in favour of hospital treatment for dental cases. At the same time efficient dental treatment in a school clinic cannot be given if the institution is hampered by want of funds, and has to carry on its work on so-called "cheap lines."

